# Modeling and Simulation of *h*GAT1: A Mechanistic Investigation of the GABA Transport Process

**DOI:** 10.1016/j.csbj.2018.12.003

**Published:** 2018-12-15

**Authors:** Sadia Zafar, Megin E. Nguyen, Ramaiah Muthyala, Ishrat Jabeen, Yuk Y. Sham

**Affiliations:** a Research Center for Modeling and Simulation (RCMS), National University of Sciences and Technology (NUST), Islamabad, Pakistan; b Department of Experimental and Clinical Pharmacology & Center for Orphan Drug Research, College of Pharmacy, University of Minnesota, Minneapolis, MN 55455, United States; c Department of Integrative Biology and Physiology, Medical School, University of Minnesota, Minneapolis, MN 55455, United States; d Bioinformatics and Computational Biology Program, University of Minnesota, United States

**Keywords:** GABA, Molecular dynamics simulations, *h*GAT1 translocation cycle, GABA transporter 1, Conformational analysis

## Abstract

Human γ-Aminobutyric acid transporter 1 (*h*GAT1) is a Na^+^/Cl^−^ dependent co-transporter that plays a key role in the inhibitory neurotransmission of GABA in the brain. Due to the lack of structural data, the exact co-transport mechanism of GABA reuptake by *h*GAT1 remains unclear. To examine the roles of the co-transport ions and the nature of their interactions with GABA, homology modeling and molecular dynamics simulations of the *h*GAT1 in the open-to-out conformation were carried out. Our study focused on the sequential preloading of Na^+^ and Cl^−^ ions, followed by GABA binding. Our simulations showed pre-loading of ions maintains the transport ready state of *h*GAT1 in the open-to-out conformation essential for GABA binding. Of the four putative preloaded states, GABA binding to the fully loaded state is most favored. Binding of Na^+^ ion to the Na1 site helps to maintain the open-to-out conformation for GABA binding as compared to the Na2 site. GABA binding to the mono-sodium or the di-sodium loaded states leads to destabilization of Na^+^ ions within their binding sites. The two most prominent interactions required for GABA binding include interaction between carboxylate group of GABA with the bound Na^+^ ion in Na1 binding site and the hydroxyl group of Y140. Overall our results support the fully loaded state as the predominate state for GABA binding. Our study further illustrates that Na^+^ ion within the Na1 site is crucial for GABA recognition. Therefore, a revised mechanism is proposed for the initial step of *h*GAT1 translocation cycle.

## Graphical abstract

**Figure:**
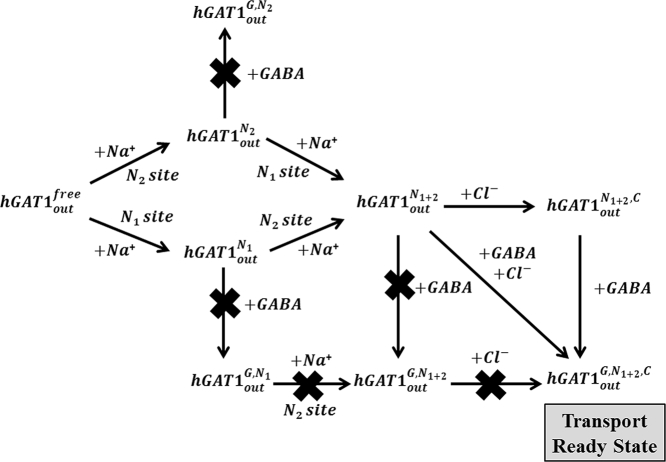

## 1 Introduction

γ-Amino butyric acid (GABA) is a major endogenous inhibitory neurotransmitter in the central nervous system (CNS) [1]. Under normal physiological condition, GABA is released from vesicles into the synaptic cleft to restore the action potential of neurons. GABA reduces neuronal excitation by binding to the GABA receptors located on the surface of the post-synaptic neuron, resulting in a concentration gradient exchange of ions and hyperpolarization of the membrane's action potential [2]. After inhibitory neurotransmission, the extracellular concentration of GABA in the synaptic cleft is restored and maintained at low level by the feedback transport mechanism of the human GABA transporter subtype 1 (*h*GAT1) and GABA transporter subtype 3 (*h*GAT3) located on the presynaptic neuron surface and nearby astrocytes [3].

**Fig. 1:**
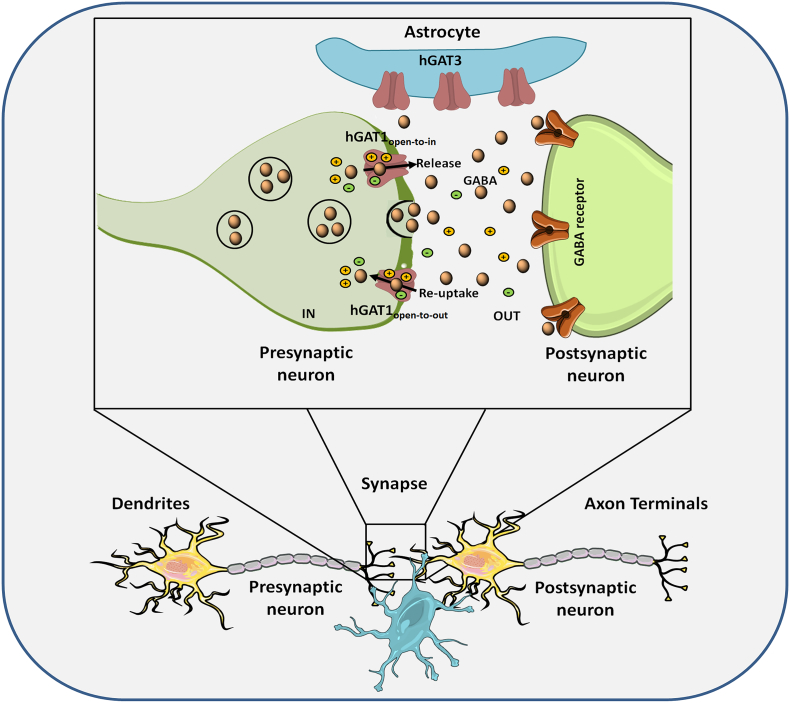
Schematic illustration of GABA release and reuptake by *h*GAT1. Inhibitory neurotransmission is mediated by vesicular release of GABA into the synaptic cleft and its binding to the GABA receptor on the postsynaptic neuron. Afterwards, excess GABA in the synaptic cleft is removed by *h*GAT1 located on the presynaptic neuron surface in the open-to-out conformation through a GABA reuptake process and by the GABA transporter subtype 3 (*h*GAT3) located on the nearby astrocytes for metabolism. Build-up of intracellular concentration of GABA in the presynaptic neuron can also lead to GABA release by *h*GAT1 in the open-to-in conformation.

*h*GAT1 functions as the primary GABA transporter in the CNS requiring two sodium and one chloride co-transport ions to facilitate the GABA translocation process. At the basal state, *h*GAT1 interconverts between “open-to-out” and “open-to-in” (*h*GAT1_open-to-out_ and *h*GAT1_open-to-in_) conformations. During the reuptake process, commonly referred as the forward mode, GABA is loaded into the *h*GAT1_open-to-out_ conformation along with the sodium and chloride ions and is co-transported from the synaptic cleft into the cytoplasm of the presynaptic neuron. Buildup of intracellular concentration of GABA within the presynaptic neuron can also lead to GABA release. In this reverse mode GABA and co-transport ions bind to the *h*GAT1_open-to-in_ conformation and are co-transported into the synapse (Fig. 1) [4,5].

Abnormal neurotransmission that prevents the buildup of synaptic GABA concentration underlies the onset of various neurological disorders such as Alzheimer's disease [6], Parkinson's disease [7], schizophrenia [8,9] and, most notably, epilepsy [10]. Inhibitory neurotransmission can be restored by inhibiting the GABA reuptake process via *h*GAT1. This prolongs the availability of GABA for binding to the GABA receptors on the postsynaptic neurons [11]. To-date, the *h*GAT1_open-to-out_ conformation involved in the GABA reuptake process has been established as a validated drug target [12] with Tiagabine as the only FDA approved drug for the treatment of epilepsy [13]. However, Tiagabine therapy has been associated with certain side effects including tremor, ataxia and sleep disorder, therefore, the quest for alternative *h*GAT1 inhibitors as potential antiepileptic agents has remained an active area of research for the past four decades [[14], [15], [16], [17]]. Structural modeling of *h*GAT1 in the open-to-out conformation and its co-transport mechanism of GABA, facilitated by Na^+^ and Cl^−^ ions, should provide the atomistic insight essential for the design of novel GABA reuptake inhibitors.

**Fig. 2:**
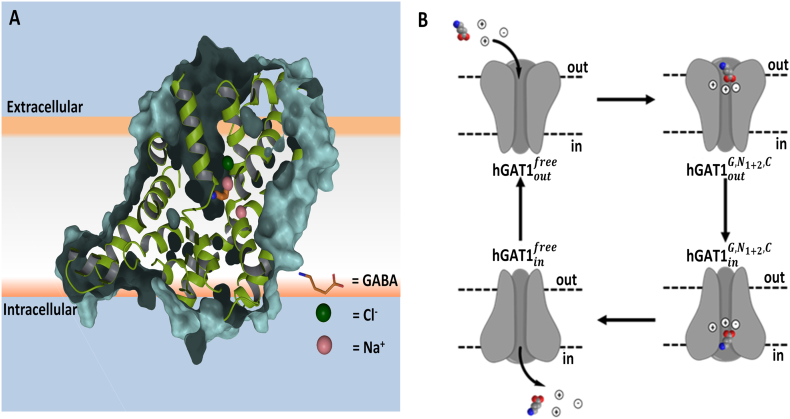
(A) Homology model of the fully loaded membrane bound *h*GAT1 complex (*hGAT*_*out*_^*G*, *N*_1+2_, *C*^) with bound GABA (G), 2 Na^+^ (N_1+2_) and 1 Cl^−^ (C) ions. (B) Schematic illustration of the GABA reuptake transport process with GABA, 2 Na^+^ and 1 Cl^−^ ions binding to the *h*GAT1 in the open-to-out conformation resulting in the formation of the *hGAT*1_*out*_^*G*, *N*_1+2_, *C*^ complex. Subsequent interconversion to the *hGAT*1_*in*_^*G*, *N*_1+2_, *C*^ conformation enables the release of the GABA and co-transport ions into the intracellular membrane as proposed by Skovstrup et al., [18], Claxton et al., [19], and Singh et al., [20].

The coupling stoichiometry of co-transport ions in the GABA translocation process remains unclear. Intense efforts have been made using the voltage clamp technique to shed light into the exact number of the sodium and chloride ions involved. Previous studies by Skovstrup et al., [18], Claxton et al., [19], and Singh et al., [20] have shown the fully loaded *h*GAT1 with two sodium and one chloride ions is required for the successful transport of GABA across the membrane (Fig. 2). However, Bicho et al., have suggested an alternative mechanism in which the Cl^−^ ion may not be required [10]. Most recently, Willford et al., have provided new evidence that the transport of GABA may involve an additional cation, most likely a sodium ion, which would result in a three Na^+^ and one Cl^−^ ions coupling stoichiometry for the GABA transport in both GAT1 and GAT3 transporters [21].

The exact mechanism of how co-transport ions facilitate the GABA transport process is not well understood. Various experimental efforts have been made to ratify the exact sequential order of ion binding required for the Na^+^/Cl^−^-dependent GABA reuptake transport process [10,22,23]. Mager et al., have hypothesized that pre-loading of Na^+^ and Cl^−^ ions is required for GABA binding [22]. Bicho et al., have reported that in the forward mode of the transport process, the binding of a single sodium ion, followed by the second sodium ion and GABA, facilitates the initialization of the GAT1 mediated GABA reuptake process [10]. Most recently, Rosenberg et al., have proposed a two steps mechanism in which the binding of two Na^+^ ions occurred, followed by the simultaneous binding of GABA and Cl^−^ ion in the subsequent step [24].

To better understand the exact mechanism of the co-transport process for GABA, homology modeling and molecular dynamics (MD) simulation of *h*GAT1_open-to-out_ conformation was carried out using the *Drosophila* dopamine transporter (*d*DAT) as the X-ray crystallographic structural template [25]. We hypothesized the maintenance of the pre-loaded state of *h*GAT1 in the open-to-out conformation is essential for GABA binding prior to GABA translocation. To examine the role of sodium and chloride ion binding during the forward mode of the GABA reuptake process, we investigated each preloaded state of the *h*GAT1. This includes the apo, mono-sodium, di-sodium and the fully loaded (two Na^+^ and one Cl^−^) states of *h*GAT1 in the presence and absence of GABA. In the absence of a high resolution X-ray crystallographic structure of a homologous transporter consisting of a third sodium ion binding site, the proposed mechanism by Willford et al., [21] was not examined in the present study. While other studies have hypothesized that Cl^−^ ion may not function as a co-transport ion [10], and is needed only to maintain the concentration gradient across the membrane [26], our study supports the widely accepted view of the fully loaded state of *h*GAT1 as the primary transport state for GABA [27].

## 2 Computational Methods

### 2.1 Homology Modeling

**Supplementary Fig. S1:**
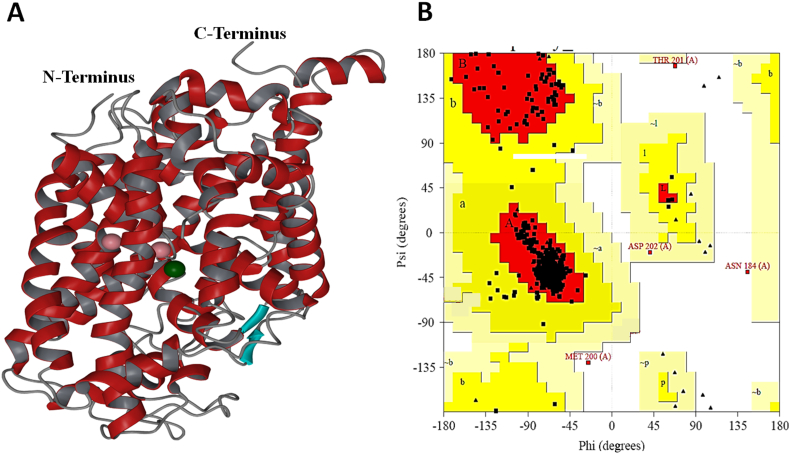
(A) Homology model of *h*GAT1 using *d*DAT (PDB ID: 4XP4) as a template. Pink spheres represent Na^+^ ions whereas green sphere represents Cl^−^ ion. (B) φ-ψ torsion angles for all the residues in the *h*GAT1 homology model.

Structural modeling was carried out using the Schrodinger modeling package [12]. Homology modeling of *h*GAT1 (UniProt: P30531) was based on the X-ray crystallographic structure of the open-to-out conformation of the *d*DAT in complex with cocaine and 2 Na^+^/1 Cl^−^ co-transport ions (PDB ID: 4XP4). The sequence alignment between *h*GAT1 and *d*DAT was based on earlier studies by Yamashita et al., [28] and Beuming et al., [29] to identify the structurally conserved regions of *h*GAT1. The final model of *h*GAT1 consists of the crystallographic bound co-transport ions taken from *d*DAT as their binding sites are highly conserved between the two homologous transporters [25]. Using the standard protein preparation protocols, the ionization states of final *h*GAT1 model was prepared at physiological pH, followed by energy minimization using OPLS 2005 force field [30] with the implicit solvent generalized Born model [31] to optimize all hydrogen-bonding networks. The final model was evaluated by PROCHECK [32] (Supplementary Fig. S1) and ERRAT [33].

### 2.2 Modeling *h*GAT1 Complexes

The fully loaded homology model of *h*GAT1 consists of GABA along with its two sodium and one chloride ions. In order to explore the exact mechanism of the GABA transport process, each of the nine putative ion-preloaded states of the *h*GAT1 in the open-to-out conformation were examined. These include the (i) *hGAT*1_*out*_^*free*^, (ii) *hGAT*1_*out*_^*N*_1_^, (iii) *hGAT*1_*out*_^*N*_2_^, (iv) *hGAT*1_*out*_^*N*_1+2_^, (v) *hGAT*1_*out*_^*N*_1+2_, *C*^, (vi) *hGAT*1_*out*_^*G*, *N*_1_^, (vii) *hGAT*1_*out*_^*G*, *N*_2_^, (viii) *hGAT*1_*out*_^*G*, *N*_1+2_^, and (ix) *hGAT*1_*out*_^*G*, *N*_1+2_, *C*^ states where G, N and C represent the bound GABA, Na^+^ and Cl^−^ ions, respectively and the N_1_, N_2_ and N_1+2_ denote the mono or the di-sodium ions bound at the Na1 and Na2 sites. The coordinates of the bound ions were taken from the structural template of *d*DAT. GABA was subsequently modeled, energy minimized in implicit solvent, and docked into the putative *h*GAT1 binding site defined by the bound cocaine substrate within the *d*DAT template using Glide [[34], [35], [36]].

### 2.3 Molecular Dynamics Simulation

Each of the *h*GAT1 complexes was embedded within 15 Å buffer region inside a box of POPC lipid membrane bilayer and explicit TIP3P water using Desmond [37]. The system was electroneutralized by 0.15 M NaCl salt concentration as counter ions. Each of the complexes was initiated by Desmond's default initialization protocol, followed by 50 ns production simulation using the OPLS 2005 force field under constant area isothermal isobaric (NPAT) conditions at 300 K and 1 atm.

The root mean square deviation of the C_α_ atoms (C_α_RMSD) from its initial coordinates and the root mean square fluctuation of the C_α_ atoms from its mean position (C_α_RMSF) serve as the reference measure for the overall stability and conformational movements of the *h*GAT1 upon co-transport ions and GABA binding. Since all the nine modeled preloaded states were based on the *h*GAT1 homology model, the evaluation of C_α_RMSD for all simulations utilized the same reference structure.

## 3 Results and Discussion

### 3.1 Homology Modeling

**Fig. 3:**
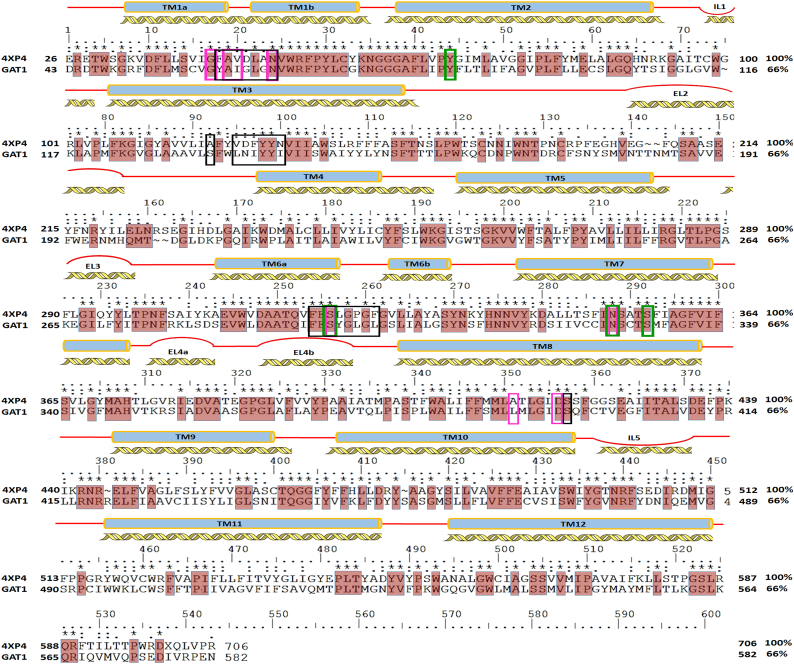
Sequence alignment of *h*GAT1 and *d*DAT. The binding site residues within 5 Å distance of GABA (black box) and the ion binding residues for the Na^+^ (pink box) and Cl^−^ (green box) ions are highlighted. The conserved residues are marked with *. Secondary structural elements and transmembrane (TM) segments are represented with yellow helices and blue bars, respectively. (For interpretation of the references to colour in this figure legend, the reader is referred to the web version of this article.)

Numerous factors such as sequence identity, functional similarity, structural resolution, and the sequence alignment are essential in determining the structural template used in homology modeling [38,39]. The latter is particularly critical for identifying structurally conserved regions, binding sites, and transmembrane regions. Here the homology modeling of *h*GAT1 (UniProt ID: P30531) in the open-to-out conformation was carried out based on the X-ray crystallographic structure of *d*DAT (PDB ID: 4XP4) in complex with its cocaine substrate and co-transport di-sodium and chloride ions. The X-ray crystallographic structural resolution between *Aquifex aeolicus* leucine transporter (*A*_*a*_LeuT) and *d*DAT was 2.0 Å and 2.8 Å, respectively. The overall sequence homology between *h*GAT1 and *d*DAT was 66% (Fig. 3), significantly higher than the 36% sequence homology of the *A*_*a*_LeuT used previously [18,28,29]. All three homologs (*h*GAT1, *d*DAT and *A*_*a*_LeuT) possess functional similarity as small molecules co-transporters, with nearly ~75% shared sequence homology defining their structurally conserved ion and substrate binding sites. This includes the highly conserved Y139, Y140, I143, W146, F294, N327 and S396 binding site residues of *h*GAT1, whose importance has been well articulated through previous mutagenesis studies [18,24,27,40]. Finally, *A*_*a*_LeuT is a Cl^−^ independent co-transporter which lacks the structurally conserved chloride ion binding site likely due to the S331E mutation [[41], [42], [43]]. As such, *d*DAT was selected as the preferred structural surrogate for the development of the revised *h*GAT1 homology model [18,28,29].

The homology model of *h*GAT1 showed the characteristic topology of 12 transmembrane (TM) helices along with the intracellular (IL) and extracellular loops (EL) (Supplementary Fig. S1A). The quality of the *h*GAT1 model was evaluated using Ramachandran plot [32] and ERRAT [33]. The Ramachandran plot displayed 93.5% of the residues of *h*GAT1 in most favored regions, 5.2% residues in additionally allowed regions, 0.4% residues in generously allowed regions, and 0.9% residues in disallowed regions (Supplementary Fig. S1B). The residues in the disallowed region include N184, M200, T201, and D202 located within the EL2. This was not surprising as the EL2 in the homologs possess less number of amino acid residues and hence was not modeled properly in *h*GAT1 [44]. Moreover, ERRAT [33] scored 82.7% hereby supporting the overall reliability of the *h*GAT1 model.

### 3.2 Molecular Dynamics Simulations

The present study explores the co-transport mechanism of *h*GAT1 by examining each of the four putative preloaded states of *h*GAT1 and its interaction with GABA. The preloaded states include the mono-sodium ion loaded state in the Na1 or Na2 site (*hGAT*1_*out*_^*N*_1_^ and *hGAT*1_*out*_^*N*_2_^), the di-sodium loaded state in both the Na1 and Na2 sites (*hGAT*1_*out*_^*N*_1+2_^), and the fully loaded state with two sodium and one chloride ions in the Na1, Na2 and Cl sites (*hGAT*1_*out*_^*N*_1+2_, *C*^). The four putative transport ready states involve each of the explored preloaded states with bound GABA (*hGAT*1_*out*_^*G*, *N*_1_^, *hGAT*1_*out*_^*G*, *N*_2_^, *hGAT*1_*out*_^*G*, *N*_1+2_^ and *hGAT*1_*out*_^*G*, *N*_1+2_, *C*^). The apo state (*hGAT*1_*out*_^*free*^) was included as the reference state for the study.

**Fig. 4:**
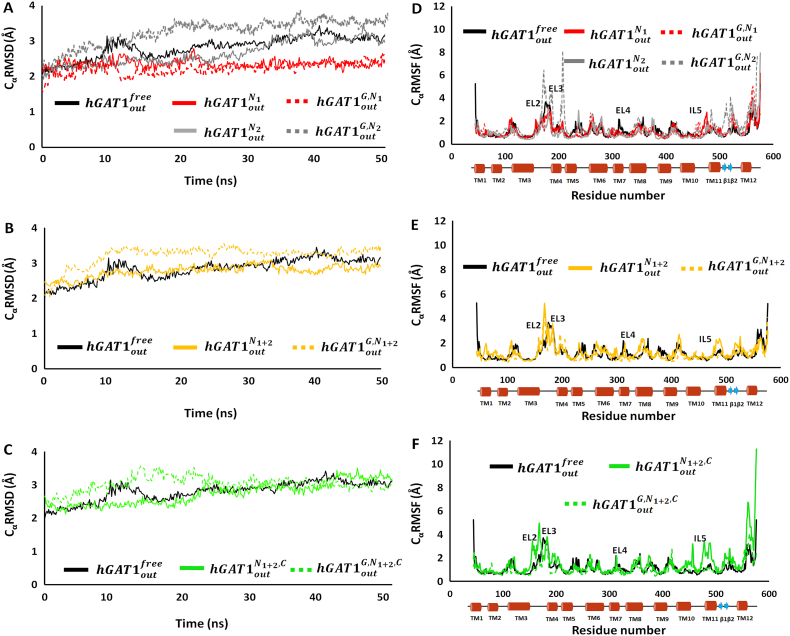
(A-C) C_α_RMSD and (D-F) C_α_RMSF plots for the apo (black), mono-sodium (red and grey), di-sodium (yellow) and the fully loaded (green) state of *h*GAT1 with (dotted line) and without (solid line) GABA. The apo state *h*GAT1_out_^free^ (black), serves as the reference state for the simulation. Overall GABA binding leads to destabilization of the *hGAT*1_*out*_^*G*, *N*_2_^ and *hGAT*1_*out*_^*G*, *N*_1+2_^ complexes with increase conformational flexibility in the EL2 region between TM3 and TM4 of *h*GAT1.

To explore the overall stability and dynamic behavior of *h*GAT1, molecular dynamics (MD) simulations were carried out for each of its putative state within a POPC phospholipid membrane bilayer with TIP3P explicit water model at 0.15 ionic salt concentration. The C_α_RMSD (A-C) and the C_α_RMSF (D-F) for each of the MD simulations are shown in Fig. 4. The reference simulation of the apo state (*hGAT*1_*out*_^*free*^) showed convergence after the first 10 ns of the simulation with an average C_α_RMSD over 2.9 Å, indicative of a stable *h*GAT1 homology model undergoing conformational optimization within the phospholipid bilayer membrane-solvent environment. Both the mono-sodium preloaded states, *hGAT*1_*out*_^*N*_1_^ and *hGAT*1_*out*_^*N*_2_^ with sodium ion bound to either the N1 or N2 site in *h*GAT1 exhibited an average C_α_RMSD of 2.4 Å and 2.9 Å, respectively (Fig. 4A). Subsequent binding of GABA to these two states resulted in an average C_α_RMSD of 2.4 Å and 3.3 Å for *hGAT*1_*out*_^*G*, *N*_1_^ and *hGAT*1_*out*_^*G*, *N*_2_^ complexes, respectively. The marked increase in the average C_α_RMSD for *hGAT*1_*out*_^*G*, *N*_2_^ over *hGAT*1_*out*_^*G*, *N*_1_^ suggests preloading of sodium ion to the Na1 site is crucial, and may be preferred over the Na2 site, for stabilizing the *h*GAT1 in the open-to-out conformation for both GABA and co-transport ion binding prior to translocation.

**Supplementary Fig. S2:**
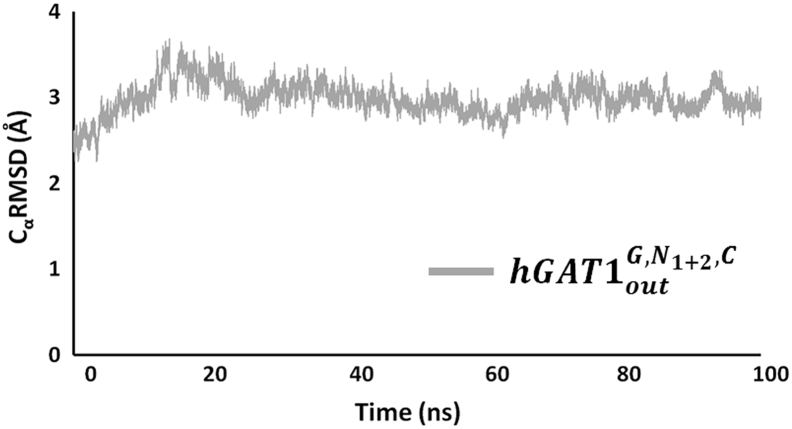
Extended MD simulation of fully loaded *h*GAT1 (*hGAT*1_*out*_^*G*, *N*_1+2_, *C*^) to 100 ns.

For the *hGAT*1_*out*_^*N*_1+2_^ complex, binding of di-sodium ions resulted in an average C_α_RMSD of 2.9 Å similar to both the reference *hGAT*1_*out*_^*free*^ and the *hGAT*1_*out*_^*N*_2_^ states. Subsequent addition of GABA to the di-sodium complex (*hGAT*1_*out*_^*G*, *N*_1+2_^) increases the average C_α_RMSD to 3.5 Å (Fig. 4B), comparable to the observed changes for GABA binding to the *hGAT*1_*out*_^*N*_2_^ state. The overall increase in the average C_α_RMSD for both the *hGAT*1_*out*_^*G*, *N*_1+2_^ and the *hGAT*1_*out*_^*G*, *N*_2_^ complexes, as compared to their unbound GABA states (*hGAT*1_*out*_^*N*_1+2_^ and *hGAT*1_*out*_^*N*_2_^), suggests neither preloaded states afford sufficient stabilization environment for GABA binding. For the *hGAT*1_*out*_^*N*_1+2_, *C*^ complex, addition of a single Cl^−^ ion to the di-sodium complex resulted in an average C_α_RMSD value of 3.0 Å (Fig. 4C). In this preloaded state, binding of GABA (*hGAT*1_*out*_^*G*, *N*_1+2_, *C*^) also resulted in an average C_α_RMSD of 3.0 Å. In contrast to the *hGAT*1_*out*_^*G*, *N*_1+2_^ and the *hGAT*1_*out*_^*G*, *N*_2_^ states, the presence of the Cl^−^ ion with the di-sodium ions helped to stabilize the overall *h*GAT1 structure both prior to and during GABA binding. Therefore, we propose the fully loaded state with the di-sodium and chloride ions as the preferred preloaded state for GABA binding required for the reuptake transport process. To validate the overall stability of the fully loaded complex (hGAT1_out_^N_1+2_, C^), its simulation was further extended to 100 ns with a sustained average C_α_RMSD of 3.0 Å (Supplementary Fig. S2).

To examine the effect of ion binding on conformational changes, we further evaluated the root mean square fluctuations of the backbone C_α_ atoms (C_α_RMSF) for all nine *h*GAT1 complexes (Fig. 4 D-F). The highest C_α_RMSF values correspond to the residues located in the hydrophilic ELs, the ILs and terminal loops. The residues in the TM segments showed the lowest values of C_α_RMSF. The highest C_α_RMSF values observed corresponded to the EL2 and EL3 at 3.4 Å and 3.7 Å, respectively. EL2 located between the TM3 and TM4 underwent major conformational shift during translocation of GABA in forward mode. It was also observed that cytoplasmic region of the TM3 become more flexible due to the unwinding over the course of simulation period. This might be a result of poor solvent-lipid interactions as it was observed in all of the *h*GAT1 proposed model states. Overall, the C_α_RMSF have shown that the ligand-protein complex of fully loaded *h*GAT1 is reasonably stable in the lipid bilayer-aqueous environment.

Binding of the di-sodium ions is required in all the three homologs of *A*_*a*_LeuT, *d*DAT and *h*GAT1, but the exact order of the sodium ions preloading process remains unclear. Bicho et al., has proposed that a single sodium ion binds first into the *h*GAT1 binding pocket, followed by other ions and substrate [10]. In order to explore Bicho's ion transport mechanism, we examine the interatomic distances between each of the sodium ions and GABA with its nearest neighboring residues in *h*GAT1 (Table 1). For the *hGAT*1_*out*_^*N*_1_^ and *hGAT*1_*out*_^*N*_2_^ complexes with the mono-sodium ion located at either the Na1 and Na2 sites in the absence of GABA, the average distances for the A61, N66, S295 and N327 residues surrounding the sodium ion at the Na1 site were 3.2 Å, 2.5 Å, 2.5 Å and 2.5 Å, respectively during the 50 ns MD simulation. For the sodium ion located at the Na2 binding site, the average distances for G59, I62, L392 and D395 were 2.7 Å, 2.4 Å, 3.1 Å and 2.3 Å, respectively. Examining the binding of GABA to the mono-sodium complex (*hGAT*1_*out*_^*G*, *N*_1_^ or *hGAT*1_*out*_^*G*, *N*_2_^) helped to identify its effect on sodium ions binding. In the *hGAT*1_*out*_^*G*, *N*_1_^ complex, binding of GABA alters the Na1 interactions with surrounding A61 and S295, resulting in an average distance of 4.4 Å and 4.9 Å while forming a direct charge-charge interaction between its negatively charged carboxylate group with the positively charged Na1 at 3.0 Å. For the *hGAT*1_*out*_^*G*, *N*_2_^ complex the average distance between Na2 with residues G59 and I62 was dramatically increased to 3.9 Å and 6.7 Å respectively. Since the GABA binding site was far away from the Na2 site, the average distance between GABA's carboxylate group to the Na2 sodium ion was 8.9 Å. This is consistent with current view that the *hGAT*1_*out*_^*G*, *N*_2_^ complex is considered as a “non-GABA reuptake/transport state” because of the absolute requirement for sodium ion interaction with GABA's carboxylate group [45].

**Table 1:** The average interatomic distances between GABA, sodium and chloride ions with its nearby binding site residues.

		GABA free			GABA bound		
*h*GAT1 Complex	Residues	Na1	Na2	Cl^−^	Na1	Na2	Cl^−^
+Na1	A61 O N66 OD1 S295 O S295 HO N327 OD1 GABA O1	3.2 2.5 2.5 3.7 2.5 -	- - - - - -	- - - - - -	4.4 2.6 2.7 4.9 2.5 3.0	- - - - - -	- - - - - -
+Na2	G59 O I62 O L392 O D395 O1 D395 O2 GABA O1	- - - - - -	2.7 2.4 3.1 2.3 3.4 -	- - - - - -	- - - - - -	3.9 6.7 3.0 2.5 2.3 8.9	- - - - - -
+Na1/Na2	A61 O N66 OD1 S295 O S295 HO N327 OD1 GABA O1 G59 O I62 O L392 O D395 O1 D395 O2	3.4 2.5 2.5 3.5 2.6 - - - - -	- - - - - - 2.5 3.2 2.8 2.6 2.4	- - - - - - - - - - -	2.5 2.5 2.8 5.6 5.1 2.5 - - - - -	- - - - - - 2.5 2.6 2.7 2.8 3.1	- - - - - - - - - - -
+Na1/Na2/Cl	A61 O N66 OD1 S295 O S295 HO N327 OD1 GABA O1 G59 O I62 O L392 O D395 O1 D395 O2 Y86 HO S295 HO N327 H1ND S331 HO	2.6 2.6 2.5 2.6 3.0 - - - - - - - - - -	- - - - - - 2.7 2.8 2.9 2.5 2.8 - - - -	- - - - - - - - - - - 3.3 4.1 3.4 3.7	2.4 2.6 2.7 2.7 2.9 2.3 - - - - - - - - -	- - - - - - 2.5 2.5 2.5 2.4 2.5 - - - -	- - - - - - - - - - - 2.3 2.6 3.5 2.4

In di-sodium*h*GAT1 complex (*hGAT*1_*out*_^*N*_1+2_^), both Na1 and Na2 ions were observed at a interatomic distance of 7.6 Å. Both sodium binding sites are separated and screened from one another by residues A61 and I62, each of which forms a direct interaction with one of the sodium ions [46,47]. The addition of GABA to the di-sodium complex (*hGAT*1_*out*_^*G*, *N*_1+2_^) resulted in an increase in the interatomic distances between Na1 with S295 to 5.6 Å and N327 to 5.1 Å while the interactions between Na1 and A61, N66, S295 were relatively preserved. The average interatomic distance between GABA's carboxylate group to Na1 in the di-sodium complex was slight lower at 2.5 Å as compared to the 3.0 Å in the *hGAT*1_*out*_^*G*, *N*_1_^ complex.

**Supplementary Fig. S3:**
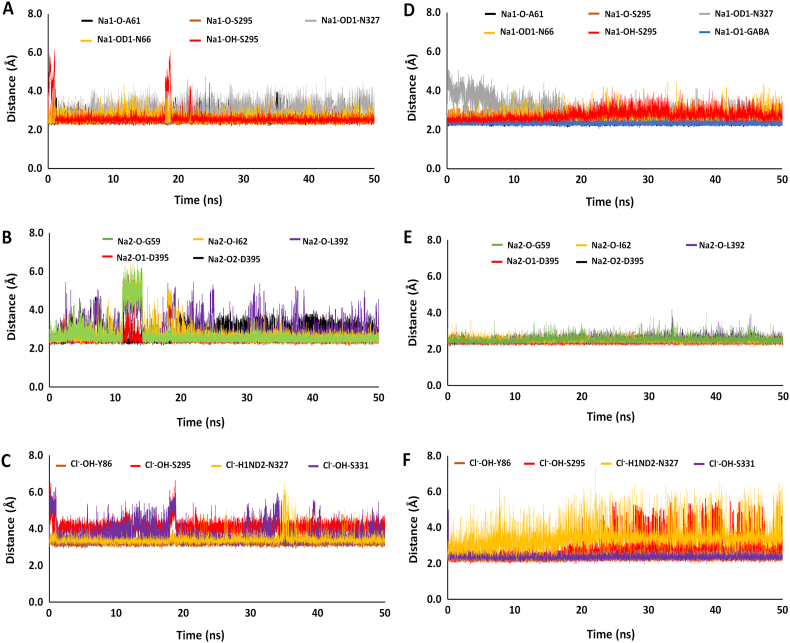
Distances between the (A) Na1 binding site residues, (B) Na2 binding site residues and (C) Cl^−^ binding site residues of *h*GAT1 over the course of 50 ns simulation time in *hGAT*1_*out*_^*N*_1+2_, *C*^ and in fully loaded complex in the presence of GABA i.e., *hGAT*1_*out*_^*G*, *N*_1+2_, *C*^ in D, E and F.

Addition of Cl^−^ ion to the di-sodium complexes in the absence of GABA (*hGAT*1_*out*_^*N*_1+2_, *C*^) showed the lowest overall interatomic distances for Na1 and Na2 with the nearby residues all between the ranges of 2.5 to 3.0 Å. The interatomic distances between Cl^−^ ion with Y86, S295, N327 and S331 were all between 3.3 and 4.1 Å. In the fully loaded *hGAT*1_*out*_^*G*, *N*_1+2_, *C*^ complex with bound GABA, the overall average interatomic distances for the ion binding site residues surrounding the Na2 and Cl^−^ ions diminishes, indicative of enhance stability and binding affinity. The interatomic distances between the ions and their respective binding site residues for the fully loaded *h*GAT1 complex with and without GABA over the course of MD simulation are shown in Supplementary Fig. S3. For the fully loaded GABA bound complex, we were able to identify six stable interactions surrounding Na1 ion, as compared to the typical four found in the *hGAT*1_*out*_^*G*, *N*_1_^ and *hGAT*1_*out*_^*G*, *N*_1+2_^ states (Table 1). The interatomic distance between GABA and Na1 was 2.3 Å, the shortest of all four simulated GABA bound complexes. Overall, our interatomic distance analysis showed similar interaction profile for mono-sodium and di-sodium binding to the Na1 and Na2 sites. The presence of a sodium ion in the Na1 site is crucial for GABA binding. The binding of the Cl^−^ ion is essential for stabilizing the di-sodium ions both in the GABA unbound and bound complex. Its presence further improves the interatomic distance between GABA and the sodium ion in the Na1 site.

**Fig. 5:**
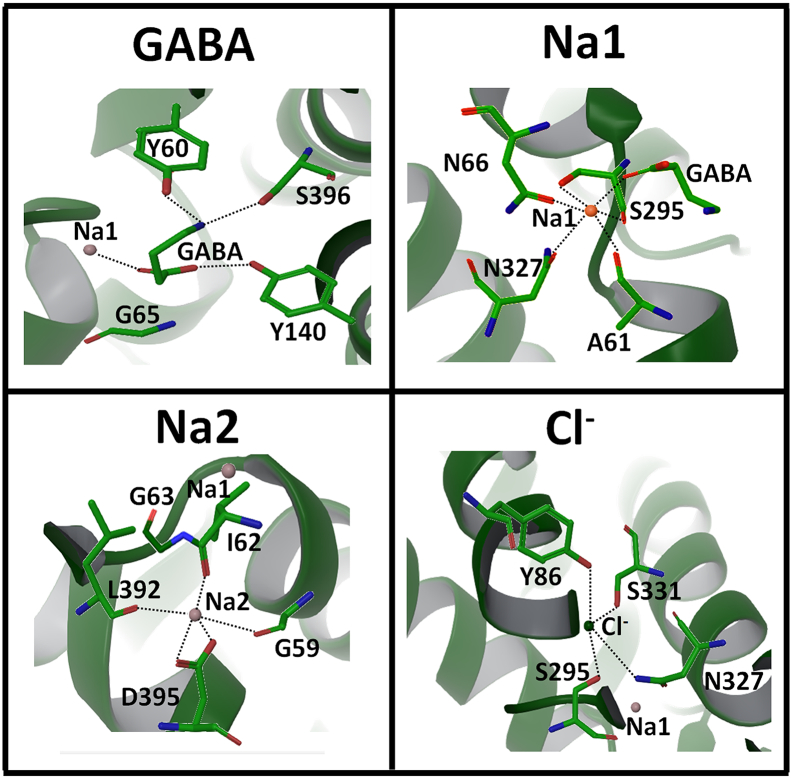
Interaction between (A) GABA, (B-C) sodium and (C) chloride ions with their nearby binding site residues in the fully loaded *h*GAT1. GABA binding involve direct interaction to the sodium ion at the Na1 site.

**Fig. 6:**
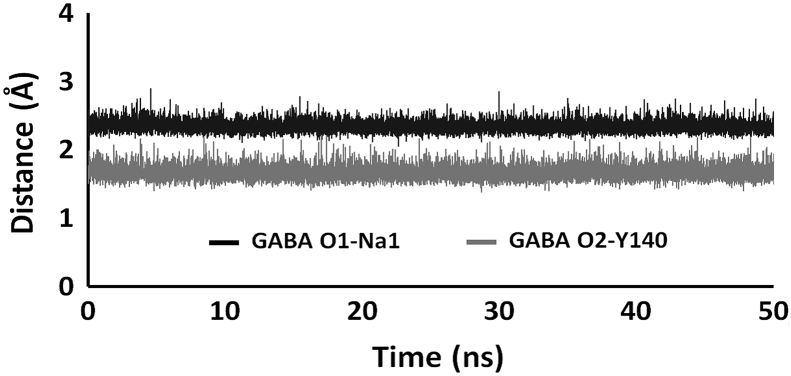
Interatomic distances between GABA carboxylate oxygen atoms to the Na^+^ ion in the Na1 site and the hydroxyl group of Y140.

Based on MD simulation and detailed analysis of the protein-ligand interactions, the fully loaded *hGAT*1_*out*_^*G*, *N*_1+2_, *C*^ state is the most promising of the four examined GABA bound *h*GAT1 complexes for GABA transport. In addition to its interaction to the Na1 ion, the GABA's carboxylate group also formed a hydrogen bonding interaction with the hydroxyl group of Y140 (Fig. 5A and Fig. 6). The importance of Y140 in *h*GAT1 has been demonstrated experimentally as a known binding determinant of GABA [48,49]. Mutation of Y140 has been shown to abolish its transport ability of GABA in the forward mode [50]. Its importance has also been explored by Skovstrup et al., with constrained MD simulation [18]. Furthermore, as a zwitterion, GABA's positively charged ammonium group also forms hydrogen bond with the hydroxyl groups of Y60 and S396. Interestingly, S396 has been shown to play a significant role in forming the lid type structure of EL4 that helps in the establishment of a hydrophobic cavity around *h*GAT1 binding site [51,52]. It has also been established that binding of the GABA requires a major conformational change in EL4 [53].

The interactions of sodium and chloride ions with the surrounding binding site residues in fully loaded *h*GAT1 were also examined. Na1 occupied its respective position in the binding pocket by interacting with the side chain atoms of N66, S295 and N327 and carbonyl oxygen of the A61 and S295 (Fig. 5B) along with electrostatic interaction with carboxylate group of GABA (Fig. 6). Yamashita et al., also observed direct interaction between the Na1 and leucine in *A*_*a*_LeuT previously [28] while it was water-mediated between the amino group of dopamine and Na1 in *d*DAT [25]. On the other hand, the surrounding residues of Na2 showed interactions with backbone atoms of G59, I62, L392 and side chain atoms of D395 (Fig. 5C). In addition, the side chain of G63 formed a bridge between Na1 and Na2 in fully loaded *h*GAT1 (Fig. 5C). The importance of G63 is well studied by Kanner in GABA permeation pathway through mutagenesis study [1]. It has been established that the mutation of G63 to either cysteine or serine abolishes the Na^+^ and Cl^−^ dependent GABA transport both in forward and backward mode [1].

The Cl^−^ ion in fully loaded *h*GAT1 was found in near proximity of Na1. The residues surrounding the Cl^−^ ion include Y86, S295, N327 and S331 as shown in Fig. 5D. Mari et al., [11], Skovstrup et al., [18] and Zomot et al., [26] demonstrated previously that the side chain of S295 forms a direct bridge between Na1 and Cl^−^ ions, thereby keeping them intact in their respective positions in *h*GAT1 binding pocket. Although, the side chains of amino acid residues surrounding all of the three co-transport ions (2 Na^+^ and 1 Cl^−^) were flexible throughout unconstrained 50 ns MD simulation, all of the ions remained consistently bound from their respective first frame position to the last frame position, demonstrating the rigor of our homology model and the stability of the fully loaded *h*GAT1.

**Fig. 7:**
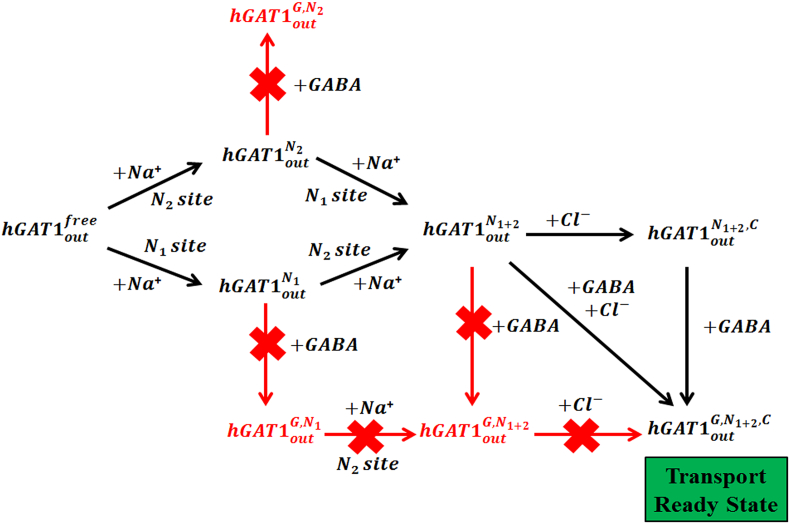
State diagram of GABA translocation cycle in open-to-out conformation of *h*GAT1. Binding of GABA to either the mono-sodium or the di-sodium bound states result in destabilization of preloaded sodium ions. The fully loaded *hGAT*1_*out*_^*G*, *N*_1+2_, *C*^ state with bound GABA provides the preferred preloaded state for the reuptake transport process within the proposed mechanistic cycle.

Based on the C_α_RMSD and inter-atomic distances between all the putative ions preloaded states of *h*GAT1, we proposed a more detailed GABA translocation cycle by *h*GAT1 (Fig. 7). According to our results, the addition of mono-sodium ion (*hGAT*1_*out*_^*N*_1_^ or *hGAT*1_*out*_^*N*_2_^) to the apo state (*hGAT*1_*out*_^*free*^) does not affect the overall stability of *h*GAT1. Formation of the di-sodium complex is also tolerable. Subsequent addition of GABA to both the mono-sodium and the di-sodium bound states resulted in the overall increase in the interatomic distances between the bound sodium ion and its neighboring residues, suggesting premature binding of GABA could lead to destabilization of the pre-loaded co-transport ions. The binding of Cl^−^ ion to di-sodium complex showed marked decrease in the overall interatomic distances between the bound sodium ions with its neighboring residues. Its presence helped to stabilize the pre-bound sodium ions within their binding sites, making the *hGAT*1_*out*_^*N*_1+2_, *C*^ the most favored state of all four co-transport ions preloaded states.

GABA binding in the fully loaded complex (*hGAT*1_*out*_^*G*, *N*_1+2_, *C*^) is the only GABA bound state with overall decrease in the interatomic distances for all pre-bound co-transport ions with their neighboring residues. It also exhibited the nearest distance between its carboxylate group with the Na1 ion relative to the GABA bound mono-sodium and di-sodium states. Simultaneous binding of GABA and Cl^−^ to the di-sodium state is possible to form the fully loaded complex. Overall, our model is consistent with the previous findings that the binding of GABA to the sodium ion is crucial for its successful transport [47,52].

## 4 Conclusion

With the availability of a high resolution X-ray crystallographic structure of dopamine transporter, homology modeling and molecular dynamics simulation of *h*GAT1 were carried out to examine the exact mechanism of the GABA transport process. Previous homology models of *h*GAT1 were based on *Aa*LeuT with only 36% sequence homology and lack a chloride ion binding site. In the current study, we have modeled and simulated the first full length homology model of *h*GAT1 based on highly homologous *d*DAT. Moreover, this is the first 50 ns molecular dynamics simulation of the full length *h*GAT1 to-date to explore multiple preloaded states (mono-sodium, di-sodium, and fully loaded) of *h*GAT1 involved in the translocation cycle of GABA.

We hypothesized that the preloaded state of *h*GAT1 in open-to-out conformation is essential for GABA binding prior to the reuptake of GABA. Our results support the fully loaded *h*GAT1 as the most favored state for GABA translocation. It further establishes the importance of sodium ion binding within the Na1 binding site for GABA recognition. Although the flipping of *h*GAT1 from open-to-out to open-to-in conformation was not examined in the present study, our revised mechanistic model for the GABA translocation cycle may provide an improve framework for understanding the initial step in the GABA reuptake process.

The following are the supplementary data related to this article.
